# Hepatitis B Virus Reactivation under Treatment with Nilotinib

**DOI:** 10.5005/jp-journals-10018-1147

**Published:** 2016-07-09

**Authors:** Tuncer Temel, Eren Gunduz, Esmira Sadigova, Hava Uskudar Teke, Safak Meric Ozgenel, Aysegul Harmanci Ozakyol

**Affiliations:** 1 Department of Gastroenterology, Eskisehir Osmangazi University, Eskisehir, Turkey; 2 Department of Hematology, Eskisehir Osmangazi University, Eskisehir, Turkey; 3 Department of Internal Medicine, Eskisehir Osmangazi University, Eskisehir, Turkey

**Keywords:** Chronic myeloid leukemia, Hepatitis B, Nilotinib, Tyrosine kinase inhibitor.

## Abstract

Hepatitis B virus (HBV) reactivation with imatinib, a tyrosine kinase inhibitor, has been reported in chronic myeloid leukemia. Nilotinib is a more potent second generation tyrosine kinase inhibitor and it inhibits the Src-family kinase LCK and hamper proliferation and function of CD8 (+) T lymphocytes. CD8 (+) T lymphocytes are the main cellular subset responsible for viral clearance in patients with HBV infection. We report a case of HBV reactivation under treatment with nilotinib. Fatal HBV reactivation is not usually related to death in chronic myeloid leukemia patients who have an expectation of longevity with well-tolerated oral drugs. Thus, screening for latent chronic HBV infections including assessment of hepatitis B surface antigen (HBsAg), antibody to hepatitis B core antigen (anti-HBc antibody) and antibody to hepatitis B surface antigen (anti-HBs), especially at countries with intermediate and high prevalence of HBsAg is warranted. Treatment with nucleoside analogs and close monitoring may be life-saving in this context.

**How to cite this article:**

Temel T, Gunduz E, Sadigova E, Teke HU, Ozgenel SM, Ozakyol AH. Hepatitis B Virus Reactivation under Treatment with Nilotinib. Euroasian J Hepato-Gastroenterol 2015;5(2):112-114.

## INTRODUCTION

Over one-third of the world’s population has been exposed to hepatitis B virus (HBV) and it is estimated that there are 350 million chronic carriers worldwide.^1,2^ Reactivation of HBV is best characterized as a virological event in which there is a sudden increase in viral replication. Frequently, there is a concomitant elevation in serum aminotransferase levels and bilirubin levels in severe cases. While HBV reactivation can also occur spontaneously, the most common clinical setting involves the use of chemotherapeutic or immunosuppressive drugs.^3^ Such agents are now often used in hematologic, gastrointestinal, rheumatic, dermatologic and pulmonary diseases as well as in transplantation.^4^ Hepatitis B virus reactivation with imatinib, a tyrosine kinase inhibitor (TKI), has been reported in chronic myeloid leukemia (CML).^5-10^ Nilotinib is a more potent second generation TKI and we report a case of HBV reactivation after switching from imatinib to nilotinib.

## CASE REPORT

A 59-year-old female patient was admitted to our hospital with elevated white blood cell count and hepatospleno-megaly in January 2014. Her medical history revealed arterial hypertension, diabetes mellitus, subtotal thy-roidectomy and cholecystectomy. She was receiving metformin 500 mg/day for diabetes mellitus and per-indopril 5 mg/day plus indapamide 1.25 mg/day for hypertension. In physical examination, thyroidectomy scar was observed and liver was 4 cm and spleen was 7 cm palpable. Hemoglobin level was 12.6 g/dl, white blood cell count was 98.7 × 109/l, absolute neutrophil count was 87.4 × 109/l and platelet count was 363 × 109/l. Biochemical parameters including renal and liver function tests were normal except glucose, uric acid and lactate dehydrogenase. According to her laboratory and peripheral blood examination she was thought to have CML. The diagnosis was confirmed by conventional cytogenetics, FISH and PCR tests. Sokal risk was calculated as 0.97 (intermediate risk). Imatinib 400 mg/day was started in February 2014. Hepatitis B surface antigen (HBsAg) positivity and diagnosis of chronic inactive hepatitis B was determined 4 years ago at a random check-up with positivity of HBsAg, hepatitis B e antigen (HBe Ag) and antibody to hepatitis B core antigen (anti-HBc) and negativity of HBV DNA. Prior to treatment with imatinib, HBsAg, HBeAg and anti HBc IgG results were positive and HBV DNA was negative. There was no recommendation for pre-emptive treatment to patients receiving TKI at that time. Also, health insurance agency did not provide prophylactic therapy for patients receiving TKA and thus prophylactic therapy was not initiated and monitoring for HBV reactivation was planned. BCR/ABL1 transcript was <1% and complete cytogenetic response was obtained 6 months after imatinib therapy. However, BCR/ABL1 transcript level began to increase progressively after sixth month and the therapy was changed to nilotinib 800 mg/day in December 2014. One week later, she complained about fatigue, anorexia, nausea and vomiting. Her physical examination revealed icteric sclera. Complete blood count was normal. Biochemical tests showed elevated alanine aminotransferase (ALT) (42 U/L, normal range 0-41 U/L), total bilirubin (4 mg/dl, normal range 0.1-1.1 mg/dl) and direct bilirubin (1.6 mg/dl, normal range 0-0.3 mg/dl) levels. Nilotinib was stopped because of grade 3 hyperbilirubinemia. Bilirubin levels were normal 1 week later but transaminase levels continued to increase (AST: 61 U/L, normal range 0-40 U/L, ALT: 68 U/L). Nilotinib was not started and she was called for a new visit 1 week later. In this visit, AST was 152 U/L and ALT was 195 U/L. She denied herbal medicine and alcohol intake. New viral hepatitis serology and polymerase chain reaction tests revealed HBsAg, HBeAg anti HBc IgG and HBV DNA positivity with a viral load of 3.14 × 105 IU/ml. Anti-HBs IgG, anti-HBe IgG, anti-HDV IgG, anti-HBc IgM, HDV antigen, anti HCV IgG and HCV RNA tests were negative. Abdominal ultrasound showed liver was 168 mm, liver parenchyma was coarse and liver contour was mildly irregular. Tenofovir was started and transaminase levels returned to normal within the first month. Nilotinib was restarted and she is going well with no clinical and laboratory abnormalities and HBV DNA levels are negative under tenofovir treatment.

## DISCUSSION

Hepatitis B virus reactivation is characterized by an abrupt rise of HBV DNA in patients with previously inactive or resolved HBV infection during or closely after chemotherapy. The current generally accepted definition of HBV reactivation following chemotherapy is the development of hepatitis with a serum ALT greater than three times the upper limit of normal or an absolute increase of 100 IU/L, associated with a demonstrable HBV DNA increase by at least 10-fold of the basal pretreatment levels.^11,12^ Hepatitis B virus reactivation not only occur in HBsAg positive patients but this may also take place in HBsAg negative patients with anti-HBc IgG positivity and/or anti-HBs IgG positivity.^13,14^

Imatinib is a TKI that blocks the ATP binding site of BCR/ABL and is currently recommended as the first line therapy for CML. Nilotinib is a second generation TKI and was designed to overcome imatinib resistance with a better efficacy.^15^ Hepatotoxicity has been reported with TKIs, however liver dysfunction usually resolves with either dose reduction or discontinuation of the drug. The mechanism of TKI-induced HBV reactivation remains unclear. *In vitro* studies have shown that imatinib can inhibit T-cell activation^16^ and proliferation.^17^ Nilotinib inhibits the Src-family kinase LCK and can hamper the proliferation and function of CD8 (+) T lymphocytes.^18,19^

Hyperbilirubinemia is a well-known side-effect of nilotinib. As increment in bilirubin levels have returned to normal grades after stopping nilotinib and did not increase despite the progressive increase in AST and ALT levels; it can be suggested that the hyperbilirubinemia might be a side effect of the treatment, the elevations of AST and ALT are also may be attributed with the hepa-totoxic effect of TKI’s. However, the progressive elevation in AST and ALT levels despite discontinuation of the drug, resolution of clinical findings, normalization of AST and ALT levels, negativity of HBV DNA levels after tenofovir treatment and the progress of bilirubin levels within normal ranges after restart of nilotinib therapy suggest us that the reason of hyperbilirubinemia and increment at aminotransferase levels may be due to reactivation of HBV.

Although pre-emptive treatment with nucleoside/ nucleotide analogs (NAs) before immunosuppressive treatment or anticancer cytotoxic chemotherapy is recom-mended,^20,21^ there was no recommendation on whether NAs should be given in patients undergoing TKI therapy till the e-publication of American Gastro-enterological Association (AGA) guidelines for the prevention and treatment of HBV reactivation during immune-suppressive drug therapy at October 2014. HBsAg positive/anti-HBc IgG positive or HBsAg negative/anti-HBc IgG positive patients treated with TKI are defined as moderate-risk group with anticipated incidence of HBV reactivation to 1 to 10% of cases and AGA suggests antiviral prophylaxis over monitoring for patients at moderate risk undergoing immune-suppressive drug therapy with weak recommendation; moderate-quality evidence.^22^ CD8 T cells are the main cellular subset responsible for viral clearance in patients with HBV infection. Tyrosine kinase inhibitor may hamper the proliferation and function of CD8 (+) T lymphocytes. Antiviral prophylaxis to patients treated with TKI (suppressors of CD8 T cells, the main cellular subset responsible for viral clearance at patients with HBV inhibitors) can be life-saving in CML patients who have an expectation of longevity with well-tolerated oral drugs.^16-19,23^

## CONCLUSION

Our case highlights the importance of HBV reactivation in CML patients receiving second generation TKIs. Fatal HBV reactivation should not be the cause of death in CML patients who have an expectation of longevity with well-tolerated oral drugs. Screening for latent chronic HBV infections with HBsAg, anti-HBc IgG, anti-HBs IgG, especially at countries with intermediate and high prevalence of HBs Ag, and pre-emptive treatment with NAs at patients with HBsAg, anti HBc IgG positivity and close follow-up might be life-saving.
